# Predicting the survival benefit of cardiac resynchronization therapy with defibrillator function for non-ischemic heart failure—Role of the Goldenberg risk score

**DOI:** 10.3389/fcvm.2022.1062094

**Published:** 2023-01-10

**Authors:** Eperke D. Merkel, Walter R. Schwertner, Anett Behon, Luca Kuthi, Boglárka Veres, István Osztheimer, Roland Papp, Levente Molnár, Endre Zima, László Gellér, Annamária Kosztin, Béla Merkely

**Affiliations:** Heart and Vascular Center, Semmelweis University, Budapest, Hungary

**Keywords:** implantable cardioverter defibrillator, cardiac resynchronization therapy, non-ischemic heart failure, risk score, sudden cardiac death

## Abstract

**Aims:**

Primary prevention of sudden cardiac death (SCD) in non-ischemic heart failure (HF) patients remains a topic of debate at cardiac resynchronization therapy (CRT) implantation requiring individual risk assessment. Using the Goldenberg SCD risk score, we aimed to predict, which non-ischemic HF patients will benefit from the addition of an implantable cardioverter defibrillator (ICD) to CRT at long-term.

**Methods:**

Between 2000 and 2018 non-ischemic HF patients undergoing CRT implantation were collected into our retrospective registry. The Goldenberg risk score (GRS) was calculated by the presence of atrial fibrillation, New York Heat Association (NYHA) class > 2, age > 70 years, blood urea nitrogen > 26 mg/dl and QRS > 120 ms. The primary endpoint was all-cause mortality, heart transplantation or left ventricular assist device implantation.

**Results:**

From 667 patients, 347 (52%) underwent cardiac resynchronization therapy-pacemaker (CRT-P), 320 (48%) cardiac resynchronization therapy-defibrillator (CRT-D) implantations. During the median follow up time of 4.3 years, 306 (46%) patients reached the primary endpoint (CRT-D 37% vs. CRT-P 63%; *p* < 0.001). CRT-D patients were younger (64 vs. 69 years; *p* < 0.001), infrequently females (26 vs. 39%; *p* < 0.001), and had a lower ejection fraction (27 vs. 29%; *p* < 0.01) compared to CRT-P patients. After GRS calculation, patients were dichotomized by low (< 3) and high (≥ 3) scores. CRT-D patients with low GRS showed a mortality benefit compared to CRT-P (HR 0.68; 95% CI 0.48–0.96; *p* = 0.03), high-risk patients did not (HR 0.84; 95% CI 0.62–1.13; *p* = 0.26).

**Conclusion:**

In our non-ischemic cohort, patients with low GRS showed a clear long-term mortality benefit by adding ICD to CRT, however, in high-risk patients no further benefit could be observed.

## Graphical Abstract

Goldenberg sudden cardiac risk score in non-ischemic heart failure patients receiving cardiac resynchronization therapy – a retrospective observational study. 
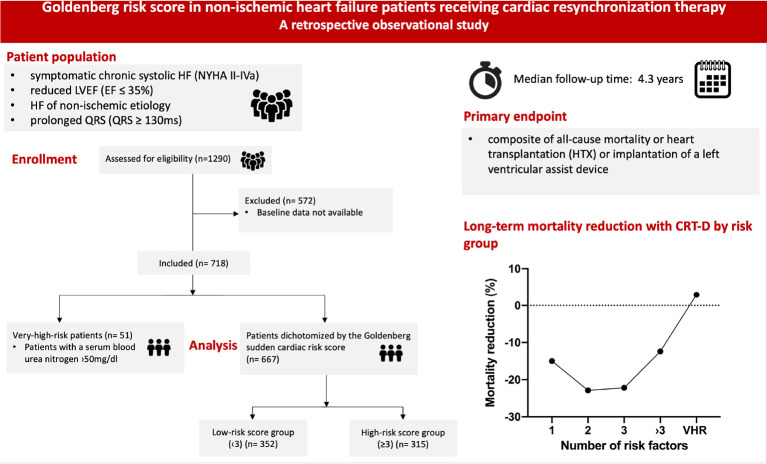

## Introduction

Cardiac resynchronization therapy (CRT) reduces morbidity and mortality in symptomatic heart failure (HF) patients with reduced left ventricular ejection fraction (LVEF) (HFrEF) and wide QRS (1).

Recommendations for the implantation of CRT devices give clear guidance to physicians in HF patients, yet its supplementation with an implantable cardioverter defibrillator (ICD) remains a topic of debate (2, 3). To choose the optimal device type, physicians must take into consideration survival modulating risk factors individually [both for sudden cardiac death (SCD) an non-SCD mediated death], such as age, etiology and the presence of fibrosis, renal dysfunction and other comorbidities, life expectancy and the preference of patients (2, 4). No randomized controlled trial was yet conducted to directly compare cardiac resynchronization therapy-defibrillator (CRT-D) to cardiac resynchronization therapy-pacemaker (CRT-P) including patients regardless of etiology. The COMPANION trial was designed to compare optimal medical therapy (OMT) to cardiac resynchronization, where CRT-D reduced mortality of any cause by 27% in HF patients of ischemic etiology and by 50% in HF patients of non-ischemic etiology compared to OMT (1). However, several observational studies could not demonstrate a mortality benefit of CRT-D devices in non-ischemic patients over CRT-P (5, 6), one of the largest observational studies by Leyva et al. proved the superiority of CRT-D therapy regardless of HF etiology (7). In the DANISH trial ICD implantation with primary prevention in non-ischemic patients did not improve survival significantly except for the subgroup of younger patients of age < 68 years, besides a significant reduction could be observed in the risk of sudden death from malignant arrhythmia (8).

As the decision of implanting CRT-D vs. CRT-P is based on individual risk assessment, more risk scores were created by independent predictors of mortality (9) or on those parameters which are proved to be relevant in the outcome from large-scale trials or registries (10, 11). In these scores, the presence of atrial fibrillation, renal function, or the severity of patients’ symptoms are the most relevant (3) regardless of the etiology.

In this study, we adopted the Goldenberg risk score (GRS), which has been originally assessed to identify those ischemic HF patients who benefit from prophylactic ICD implantation mid- and long-term using the MADIT-II trials’ cohort (12, 13). We aimed to predict a specific patient population of non-ischemic etiology based on the GRS who will acquire survival benefit from a CRT-D device. Based on our hypothesis, using this simple risk score we can also identify those who can benefit the most from ICD implantation among non-ischemic CRT patients.

## Materials and methods

### Study population and evaluations

Altogether 1,290 HF patients with non-ischemic etiology underwent CRT implantation between June 2000 and September 2018 at the Heart and Vascular Centre of Semmelweis University. Indication for CRT implantation was set up based on current European Society of Cardiology (ESC) guidelines (symptomatic HF patients on optimal medical treatment, LVEF < 35% and QRS > 130 ms) (2). Data was collected retrospectively into our “Biobankok” registry. Gathered data included medical history, clinical and echocardiographic parameters, laboratory tests and parameters of the procedures. The study complies with the declaration of Helsinki and was approved by the Regional and Institutional Committee and Research; No. 161-0/2019.

### Calculation of the Goldenberg risk score

First, we treated separately those, whose se-BUN exceeded 50 mg/dl as very-high-risk (VHR) patients as previously defined at the GRS calculation (12). The risk score comprises five clinically relevant factors [serum blood urea nitrogen (se-BUN > 26 mg/dl, QRS > 120 ms, age > 70, atrial fibrillation, New York Heat Association (NYHA) > II)]. A VHR patient population has been identified and excluded (patients with a se-BUN > 50 mg/dl) as per the original article.

Besides these patients, 667 had every data available to assess their GRS, 347 underwent CRT-P and 320 underwent CRT-D implantation. The GRS was se-BUN > 26 mg/dl and QRS > 120 ms, each counted one point, ranging between 1 and 5. No patients had a 0 score since each patient had a QRS wider than > 130 ms.

After the assessment of the GRS, the total patient cohort was further dichotomized into low (< 3) and high (≥ 3) score groups. This cut-off was set as per the original article respecting that in our CRT cohort, each patient had at least 1 point during the calculation.

### Endpoints

Our primary composite endpoint was all-cause mortality, heart transplantation (HTX) or left ventricular assist device (LVAD) implantation, whichever occurred first. The exact date of death was retrieved *via* the National Health Insurance Fund of Hungary, updated in September 2019.

### Procedures

Device implantations were performed under X-ray, anteroposterior, left anterior oblique and right anterior oblique views were obtained. Leads were introduced through the cephalic or subclavian veins. Right ventricular leads were fixed dominantly into a septal position. In case of permanent atrial fibrillation, right atrial leads were not implanted. The optimal coronary sinus side branch was chosen by venogram routinely, leads were preferred to be implanted into the lateral or posterolateral vein. Left ventricular lead implantations, if failed by the coronary sinus, were carried out by epicardial or transseptal approach. Electrical parameters were evaluated intraoperatively. Implanting physicians chose the type of device following the recommendations of current guidelines, while taking into consideration the patient’s preference, age, sex, renal function, frailty, and other co-morbidities.

### Statistical analysis

Statistical analysis was performed using GraphPad Prism, version 8.4.2 (GraphPad Software, San Diego, CA, USA) and IBM SPSS Statistics, version 26 (IBM Corp., Armonk, NY, USA). Baseline characteristics, continuous variables are described as mean ± standard deviation or median and interquartile range (25th–75th percentile), as appropriate after Shapiro–Wilk normality test. Categorical data are described as counts and frequency. Variables of the subgroups were compared by using unpaired *t*-test for normal and Mann–Whitney test for non-normal continuous variables and the χ^2^ test for dichotomous variables. Time-to-event data were analyzed by log-rank test and multivariate Cox regression analysis. A *P*-value of less than 0.05 was considered statistically significant.

## Results

### Baseline clinical characteristics

Altogether 718 non-ischemic CRT patients had all the necessary baseline data available to assess the GRS, 381 (53%) patients underwent a CRT-P and 337 (47%) patients CRT-D implantation. The characteristics of the included patients and the total of 1,290 patients underwent CRT implantation are summarized in Supplementary Table 1. From the total patient cohort, 51 (8%) patients had a > 50 mg/dl se-BUN level who represented the VHR group. From the remaining group of 667 patients, 347 (52%) had CRT-D and 320 (48%) CRT-P devices. After assessing the score and dichotomization 352 (53%) patients had a low risk (GRS 1–2) and 315 (47%) had a high-risk score (GRS ≥ 3) (Supplementary Table 2).

Of all non-ischemic patients without VHR group, those with a CRT-D were significantly younger than CRT-P implanted patients (64 vs. 69 years; *p* < 0.001) and female sex was non-dominant (26 vs. 39%; *p* < 0.001), respectively. They presented with a lower [LVEF (27 vs. 29%; *p* < 0.01)]. More patients were able to take optimal medical treatment compared to those with a CRT-P at baseline (Table 1).

**TABLE 1:** Baseline clinical characteristics of the total patient cohort without very-high-risk (VHR) patients by device type.

Characteristics	Total patients *n* = 667	CRT-P *n* = 347	CRT-D *n* = 320	*P*-value
Age, years (median, 25th–75th percentile)	66 (59–73)	69 (61–75)	64 (57–71)	<**0**.**001**
Female sex, *n* (%)	219 (33)	136 (39)	83 (26)	<**0**.**001**
Ejection fraction, % (median, 25th–75th percentile)	28 (23–32)	29 (24–34)	27 (23–30)	<**0**.**01**
QRS duration, ms (median, 25th–75th percentile)	160 (140–170)	160 (140–172)	160 (140–170)	0.69
NYHA I, *n* (%)	8 (1)	2 (0.5)	6 (2)	0.16
NYHA II, *n* (%)	289 (43)	137 (39)	152 (47)	**0**.**04**
NYHA III, *n* (%)	296 (44)	161 (46)	135 (42)	0.27
NYHA IV, *n* (%)	75 (11)	47 (13)	28 (9)	0.05
Hypertonia, *n* (%)	471 (71)	250 (72)	221 (69)	0.39
Atrial fibrillation, *n* (%)	253 (38)	134 (39)	119 (37)	0.70
COPD, *n* (%)	105 (16)	70 (20)	35 (11)	<**0**.**01**
Creatinine, mg/dl (median, 25th–75th percentile)	1.0 (0.9–1.3)	1.0 (0.9–1.3)	1.0 (0.8–1.3)	0.99
BUN, mg/dl (median, 25th–75th percentile)	21.6 (16.8–27.7)	21.8 (16.8–28.3)	21.3 (16.9–27.3)	0.38
ACE-I/ARB, *n* (%)	589 (88)	297 (86)	292 (91)	**0**.**02**
Beta-blocker, *n* (%)	576 (86)	289 (83)	287 (90)	**0**.**02**
MRA, *n* (%)	445 (67)	213 (61)	232 (72)	<**0**.**01**
Loop diuretic, *n* (%)	501 (75)	263 (76)	238 (74)	0.67
Digoxin, *n* (%)	138 (21)	89 (26)	49 (15)	<**0**.**01**
Amiodarone, *n* (%)	167 (25)	67 (19)	100 (31)	<**0**.**001**
**Mortality**				
Absolute rate, *n* (%)	306 (46)	194 (56)	112 (35)	<**0**.**001**

ACE-I, angiotensin-converting enzyme inhibitor; ARB, angiotensin II receptor blocker; BUN, blood urea nitrogen; COPD, chronic obstructive pulmonary disease; CRT-D, cardiac resynchronization therapy-defibrillator; MRA, mineralocorticoid receptor antagonist; NYHA, New York Heart Association.

Bold *p*-values mean that they are significant.

Low-risk patients with a CRT-D device were significantly younger (61.3 vs. 64.1 years; *p* < 0.001) than those with a CRT-P. They presented with a lower LVEF (26.7 vs. 29.1%; *p* < 0.01), respectively. Hypertension (73 vs. 60%; *p* = 0.01) and COPD (20 vs. 9%; *p* < 0.01) were more common in the CRT-P treated group. Regarding optimal treatment, apart from mineralocorticoid receptor antagonist (MRA), the two groups were treated comparably. CRT-D implanted patients were more likely to be treated with amiodarone (30 vs. 17%; *p* < 0.001) (Table 2).

**TABLE 2:** Baseline characteristics of patients with Goldenberg risk score (GRS) < 3.

Characteristics	CRT-P *n* = 169	CRT-D *n* = 183	*P*-value
Age, years (median, 25th–75th percentile)	64.1 (58.4–70.2)	61.3 (53.8–66.8)	< **0.001**
Female sex, *n* (%)	66 (42)	56 (28)	0.09
Ejection fraction, % (mean, SD)	29.1 (7.7)	26.7 (5.5)	< **0.01**
QRS duration, ms (median, 25th–75th percentile)	155 (130–170)	160 (140–170)	0.59
NYHA I, *n* (%)	2 (1)	6 (3)	0.18
NYHA II, *n* (%)	102 (60)	116 (63)	0.09
NYHA III, *n* (%)	49 (29)	51 (28)	0.81
NYHA IV, *n* (%)	16 (9)	10 (5)	0.15
Hypertonia, *n* (%)	123 (73)	111 (60)	**0.01**
Atrial fibrillation, *n* (%)	26 (15)	28 (15)	0.98
COPD, *n* (%)	34 (20)	17 (9)	< **0.01**
Creatinine, mg/dl (median, 25th–75th percentile)	0.95 (0.85–1.14)	0.96 (0.8–1.2)	0.88
BUN, mg/dl (median, 25th–75th percentile)	19.0 (15.1–22.7)	18.8 (15.4–23.5)	0.65
ACE-I/ARB, *n* (%)	149 (88)	167 (91)	0.34
Beta-blocker, *n* (%)	144 (85)	168 (92)	0.05
MRA, *n* (%)	103 (61)	134 (73)	**0.01**
Loop diuretic therapy, *n* (%)	120 (71)	124 (68)	0.51
Digoxin therapy, *n* (%)	33 (19)	29 (15)	0.36
Amiodarone, *n* (%)	28 (16)	47 (26)	**0.04**
**Mortality**			
Absolute rate, *n* (%)	79 (47)	50 (27)	< **0.001**

ACE-I, angiotensin-converting enzyme inhibitor; ARB, angiotensin II receptor blocker; BUN, blood urea nitrogen; COPD, chronic obstructive pulmonary disease; CRT-D, cardiac resynchronization therapy-defibrillator; MRA, mineralocorticoid receptor antagonist; NYHA, New York Heart Association.

Bold *p*-values mean that they are significant.

Regarding the high-risk group, fewer female patients were implanted a CRT-D device than CRT-P (15 vs. 31%; *p* = 0.03). As in the low-risk group, CRT-D implanted patients were younger (70.8 vs. 72.2 years; *p* = 0.02). They had comparable LVEF (28 vs. 28%; *p* = 0.33) with high-risk CRT-P patients (Table 3).

**TABLE 3:** Baseline characteristics of patients with Goldenberg risk score (GRS) ≥ 3.

Characteristics	CRT-P *n* = 178	CRT-D *n* = 137	*P*-value
Age, years (median, 25th–75th percentile)	72.2 (65.6–77.4)	70.8 (62.9–75.1)	**0.02**
Female sex, *n* (%)	70 (39)	27 (19)	< **0.001**
Ejection fraction, % (median, 25th–75th percentile)	28.0 (23.0–34.0)	28.0 (22.0–31.0)	0.33
QRS duration, ms (median, 25th–75th percentile)	160 (140–179)	160 (140–170)	0.32
NYHA I, *n* (%)	0 (0)	0 (0)	> 0.99
NYHA II, *n* (%)	35 (20)	36 (26)	0.16
NYHA III, *n* (%)	112 (63)	83 (60)	0.67
NYHA IV, *n* (%)	31 (17)	18 (13)	0.30
Hypertonia, *n* (%)	127 (71)	110 (80)	0.07
Atrial fibrillation, *n* (%)	108 (60)	91 (66)	0.29
COPD, *n* (%)	36 (20)	18 (13)	0.09
Creatinine, mg/dl (median, 25th–75th percentile)	1.14 (0.93–1.51)	1.16 (0.98–1.46)	0.55
BUN, mg/dl (mean, SD)	27.3 (9.4)	26.7 (8.9)	0.56
ACE-I/ARB, *n* (%)	148 (83)	125 (91)	**0.03**
Beta-blocker, *n* (%)	145 (81)	119 (87)	0.20
MRA, *n* (%)	110 (62)	98 (71)	0.07
Loop diuretic, *n* (%)	143 (80)	114 (83)	0.43
Digoxin, *n* (%)	56 (31)	20 (16)	< **0.001**
Amiodarone, *n* (%)	39 (22)	53 (39)	< **0.01**
**Mortality**			
Absolute rate, *n* (%)	114 (64)	62 (45)	< **0.001**

ACE-I, angiotensin-converting enzyme inhibitor; ARB, angiotensin II receptor blocker; BUN, blood urea nitrogen; COPD, chronic obstructive pulmonary disease; CRT-D, cardiac resynchronization therapy-defibrillator; MRA, mineralocorticoid receptor antagonist; NYHA, New York Heart Association.

Bold *p*-values mean that they are significant.

### Primary endpoints

**FIGURE 1:**
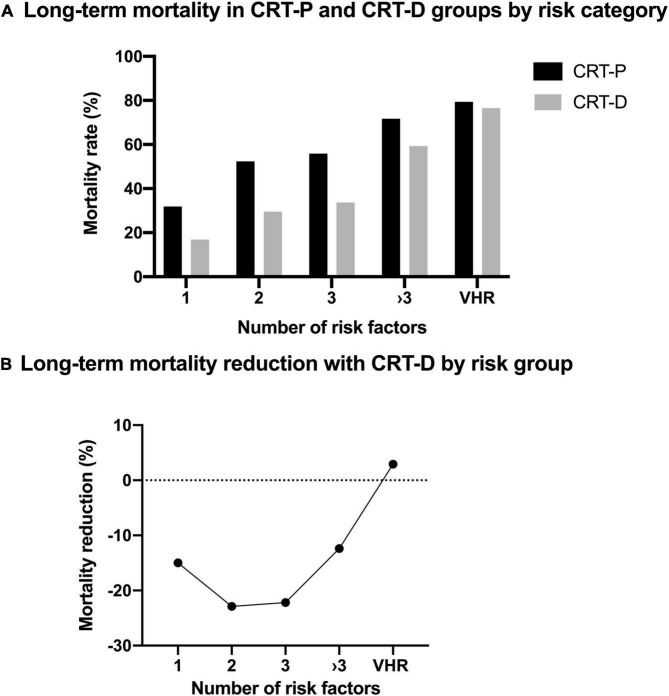
**(A)** Long-term mortality in cardiac resynchronization therapy-pacemaker (CRT-P) and cardiac resynchronization therapy-defibrillator (CRT-D) groups by risk category. Higher long-term absolute mortality rates can be observed in patients with CRT-P devices compared to CRT-D nonetheless of their risk score, except in the very-high-risk (VHR) patient population. **(B)** U-shaped curve for implantable cardioverter defibrillator (ICD) efficacy. No significant effect of CRT-D implantation can be observed in high-risk (score ≥ 3) and VHR patients. The greatest mortality reduction can be seen in low-risk patients.

Of all patients 306 (46%) reached the primary composite endpoint, 112 (37%) underwent CRT-D, 194 (63%) underwent CRT-P implantation. Our median follow-up time was 4.3 years. Regarding absolute mortality rates, fewer patients died with CRT-D therapy than with CRT-P (35 vs. 56%, *p* < 0.001), respectively (Table 1). Higher long-term absolute mortality rates can be observed in patients with CRT-P devices compared to CRT-D nonetheless of their risk score, except in the VHR patient population (Figure 1A and Supplementary Table 3). A U-shaped curve can be drawn for ICD efficacy, no significant effect of CRT-D implantation can be observed in high-risk and VHR patients. The greatest reduction of the primary composite endpoint can be seen in patients of a 2 and 3 risk score (Figure 1B).

**FIGURE 2:**
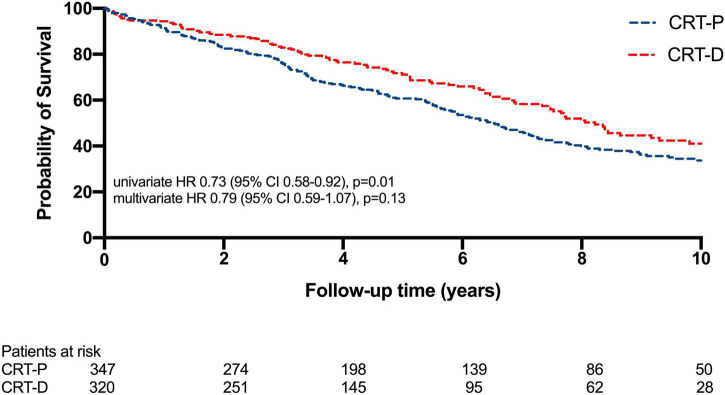
Kaplan–Meier estimates of survival comparing cardiac resynchronization therapy-defibrillator (CRT-D) and cardiac resynchronization therapy-pacemaker (CRT-P) therapies in non-ischemic patients. In the study population, at long-term a statistically significant benefit can be observed toward CRT-D compared to CRT-P therapy (HR 0.73; 95% CI 0.58–0.92; *p* = 0.01), yet at multivariate analysis this significance vanished (HR 0.79; 95% CI 0.59–1.07; *p* = 0.13).

In the total cohort without VHR group, at long-term a statistically significant benefit can be observed toward CRT-D compared to CRT-P therapy (HR 0.73; 95% CI 0.58–0.92; *p* = 0.01) (Figure 2), however by multivariate analysis, it was not confirmed (HR 0.79; 95% CI 0.59–1.07; *p* = 0.13). Cox regression analysis was adjusted for relevant clinical covariates such as age, gender, LVEF, NYHA functional class, serum urea, presence of atrial fibrillation, diabetes, hypertension, and body mass index.

When the primary endpoint was examined by the GRS, in absolute rates CRT-P implanted patients seem to have a less favorable survival rate, in low-risk patients (CRT-P 47% vs. CRT-D 27%; *p* < 0.001) and in high-risk patients (CRT-P 64% vs. 45%; *p* < 0.001).

**FIGURE 3:**
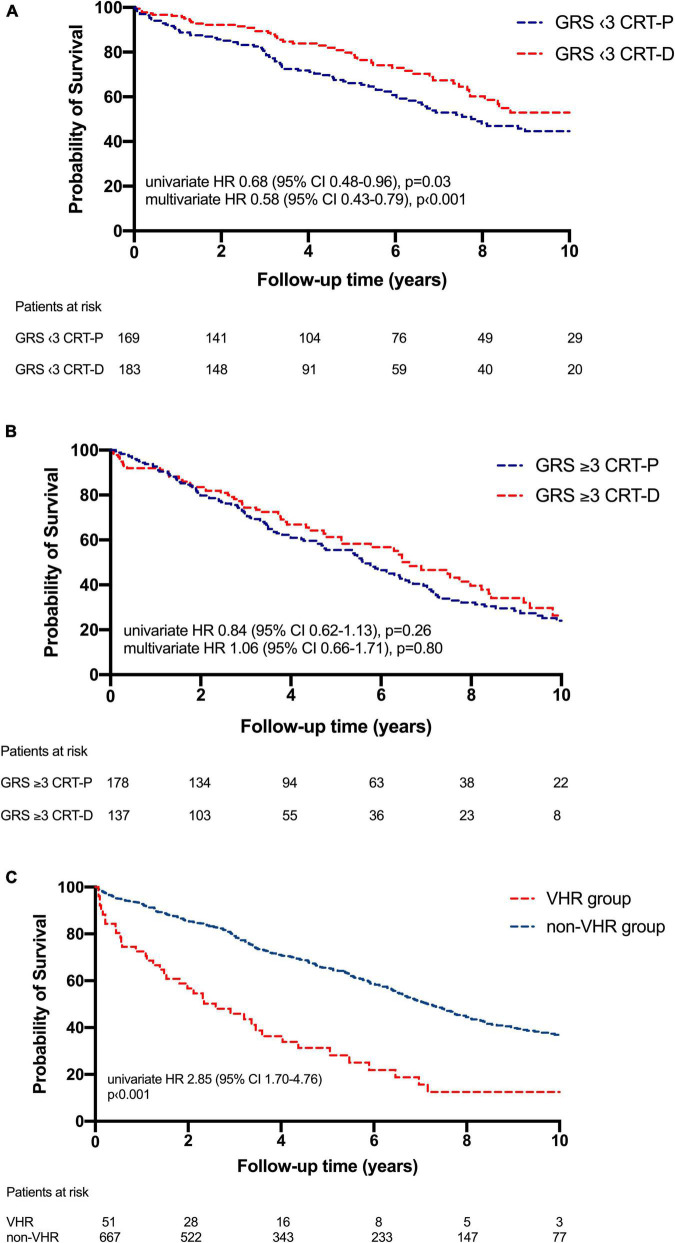
**(A)** Kaplan–Meier estimates of survival comparing cardiac resynchronization therapy-defibrillator (CRT-D) and cardiac resynchronization therapy-pacemaker (CRT-P) therapies in low-risk patients (< 3). In low-risk groups CRT-D was associated with mortality benefit at long-term (HR 0.68; 95% CI 0.48–0.96; *p* = 0.03) compared to CRT-P. **(B)** Kaplan–Meier estimates of survival comparing CRT-D and CRT-P therapies in high-risk patients [Goldenberg risk score (GRS) ≥ 3]. In this patient group CRT-D did not provide a mortality benefit (HR 0.84; 95% CI 0.62–1.13; *p* = 0.26) compared to CRT-P at long-term. **(C)** Kaplan–Meier estimates of survival in very-high-risk patients (VHR group) compared to the study population (non-VHR group). An almost threefold higher risk of all-cause mortality could be observed in the VHR patient group (HR 2.85; 95% CI 1.70–4.76; *p* < 0.001) compared to the non-VHR group.

A survival benefit could be observed with CRT-D implanted low-risk patients (risk score of 1–2) (HR 0.68; 95% CI 0.48–0.96; *p* = 0.03) compared to low-risk CRT-P treated individuals (Figure 3A). However, patients with a high-risk score (risk score ≥ 3) did not benefit from the addition of an ICD to CRT at long-term (HR 0.84; 95% CI 0.62–1.13; *p* = 0.26) (Figure 3B).

These results were also proved by Cox regression analysis. In low-risk patients CRT-D could be associated with a 42% mortality benefit (HR 0.58; 95% CI 0.43–0.79; *p* < 0.001) compared to CRT-P, not observed in high-risk patients (HR 1.06; 95% CI 0.66–1.71; *p* = 0.80) after adjusting for age, NYHA class, se-BUN, atrial fibrillation, gender and LVEF.

### Very-high-risk patient population

We identified 51 patients whose se-BUN exceeded 50 mg/dl. These VHR patients differed significantly from non-VHR patients in renal function (se-BUN 63 mg/dl vs. 21.6 mg/dl; *p* < 0.001), while atrial fibrillation was more frequent (65 vs. 38%; *p* < 0.001), CRT-D device implantations occurred in a lower number in the VHR group compared to the non-VHR group (33 vs. 48%; *p* = 0.04) (Table 4).

**TABLE 4:** Baseline characteristics comparing the non- and very-high-risk (VHR) patient groups.

Characteristics	non-VHR group *n* = 667	VHR group *n* = 51	*P*-value
BUN, mg/dl (median, 25th–75th percentile)	21.6 (16.8–27.7)	63 (55.2–71.1)	**< 0.001**
Creatinine, mg/dl (median, 25th–75th percentile)	1.0 (0.8–1.3)	2.1 (1.7–2.8)	**< 0.001**
Age, years (mean, SD)	65.6 ± 10.6	67.4 ± 9.9	0.26
Ejection fraction, % (median, 25th–75th percentile)	28 (23–32)	28 (25–30)	0.66
QRS duration, ms (median, 25th–75th percentile)	160 (140–170)	160 (140–190)	0.33
CRT-D device, *n* (%)	320 (48)	17 (33)	**0.04**
Atrial fibrillation, *n* (%)	253 (38)	33 (65)	< **0.001**
Female sex, *n* (%)	219 (33)	14 (27)	0.43
Loop diuretic therapy, *n* (%)	501 (75)	43 (84)	0.14
Digitalis therapy, *n* (%)	138 (21)	10 (20)	0.85
**Mortality**			
Absolute rate, *n* (%)	306 (46)	40 (78)	**< 0.001**

BUN, blood urea nitrogen; CRT-D, cardiac resynchronization therapy-defibrillator.

Bold *p*-values mean that they are significant.

Very-high-risk patients showed a higher absolute mortality rate (78 vs. 46%; *p* < 0.001) compared to the non-VHR group and an almost threefold higher risk of the primary endpoint by univariate analysis (HR 2.85; 95% CI 1.70–4.76; *p* < 0.001). In this selected patient group, no benefit of the ICD could be proven (HR 0.92; 95% CI 0.48–1.77; *p* = 0.81) (Figure 3C), even after adjusting for relevant covariates such as age, gender, and LVEF (HR 0.59; 95% CI 0.20–1.68; *p* = 0.32).

## Discussion

Our single center, large-scale, real-world clinical data is the first analysis with solely non-ischemic CRT patients, which used the GRS to identify the subgroup of patients who show long-term mortality benefit with adding an ICD to CRT. We found that CRT-D implantation in the total cohort of non-ischemic patients did not provide a long-term all-cause mortality difference compared to CRT-P. However, using the GRS, in a selected subgroup CRT-D implantation was beneficial, particularly in those with low and moderate risk showing a U-shaped curve for the total cohort.

Although the guideline concerning primary prevention of SCD states ICD implantation is recommended for symptomatic HF patients of non-ischemic etiology with a IB level of evidence (2), ever since the publication of the DANISH trial results, physicians’ attitude toward device implantation changed (14). The diagnosis of non-ischemic cardiomyopathy favored implanting CRT-P devices in 32% of surveyed centers, while the trial highlighted the relevance of adding an ICD regarding all-cause mortality in the subgroup of patients younger than 68 years (HR 0.64; 95% CI 0.45–0.90; *P* = 0.01). In a subgroup analysis of the COMPANION trial, a reduction in SCD was confirmed without any benefit in total mortality (1). The DEFINITE trial also discarded the benefit of adding an ICD to oral standard medical care in non-ischemic patients with respect to death from any cause (HR 0.65; 95% CI 0.40–1.06; *p* = 0.08) (15). In line with these results, more observational large-scale studies conducted by Leyva and one at our center demonstrated that CRT-D was not associated with a mortality benefit in non-ischemic patients (5, 6).

These data all confirm that the decision about adding an ICD to CRT in non-ischemic patients is still challenging and multifactorial (14). Moreover, using the current four-pillar medical treatment for HFrEF patients, adding the ICD in the elderly requires further investigation and patient-level individual assessment. For this purpose, several risk scores were attempted to assess the odds of SCD and cardiovascular mortality after CRT implantation (9, 16). Also, CRT itself reduces the risk of SCD and significantly reduces the occurrence of ventricular arrhythmias due to reverse remodeling (17). These interacting phenomena also highlight the relevance of individual risk assessment using such SCD or all-cause mortality calculators (18). An ongoing randomized controlled trial, the RESET-CRT trial is set to determine the effect of CRT-D on all-cause mortality and SCD in HF patients with CRT indication (19).

Initially, the GRS has been established to ameliorate patient selection for ICD implantation in ischemic cardiomyopathy, to outline subgroups that correspond with ICD efficacy (12). We applied the risk stratification unconventionally in non-ischemic HF patients undergoing CRT implantation, it is based on the five most relevant mortality predictors (age, atrial fibrillation, NYHA functional class, QRS width, and serum blood urea nitrogen).

Long-term outcome of non-ischemic patients with CRT-D is influenced by several parameters. The characteristics of the investigated patient cohort are essential regarding co-morbidities and the subsequent responder status. Our patient population was similarly aged [CRT-D 64 (57–71) years and CRT-P 69 (61–75) years] compared to the population in the study conducted by Barra et al. (CRT-D 66 years and CRT-P 69.8 years) (18) and to those in the MADIT-CRT trial (65 11 years) (20).

Our real-world data also shows that a selection bias can be presumed since CRT-P patients were older, with fewer females and had a higher LVEF at baseline compared to CRT-D implanted patients which may influence their outcome in CRT response and SCD rate. Similar sex distribution was observed by Barra et al. showing that fewer female patients undergo CRT implantation (33% were females in the COMPANION trial and 26% in the MADIT-CRT trial). Regarding atrial fibrillation, in the MADIT II trial, atrial fibrillation occurred less frequently compared to our study population (37 vs. 39%), however, we assessed both the current and previous events in order to involve those, who are definitely showing a higher risk to cardiovascular mortality compared to those with sinus rhythm (21).

At illustration, mortality reduction outlines as a U-shaped curve emphasizing that mostly intermediate-risk (risk factors of 2 and 3) patients benefit from CRT-D implantation (12).

The absolute mortality rates are comparable to the original article (12), 16% in non-VHR patients vs. 15.7% in our data. In the mid-term analysis of the GRS, published by Goldenberg et al. patients with an intermediate-risk gained the largest benefit from ICD therapy whereas patients with low- or high-risk did not (12). The risk stratification was also studied at long-term low-risk patients did have a significantly higher survival rate with ICD therapy than usual clinical care (HR 0.52; 95% CI 0.38–0.73; *p* < 0.001); high-risk patients with multiple comorbidities still did not acquire survival benefit (HR 0.84; 95% CI 0.63–1.13; *p* = 0.247) (13). Barra et al. enrolled patients regardless of their etiology that involved CRT candidates from a long timeframe between 2000 and 2011 and observed similar results. Patients with a low-risk score were more likely to benefit from the defibrillator, moreover, this benefit was most dominant in the first few years (11.3 vs. 24.7%, *p* = 0.041) then attenuated at long-term (21.2 vs. 32.7%, *p* = 0.078). At multivariate analysis, CRT-D decreased mortality rates compared to CRT-P (HR 0.339; 95% CI 0.178–0.642; *p* = 0.001), also seen after propensity score matching (CRT-D 20% vs. 38.2% CRT-P; *p* = 0.036) (18). In our analysis low-risk CRT-D patients showed a mortality benefit compared to CRT-P (HR 0.68; 95% CI 0.48–0.96; *p* = 0.03), whereas high-risk patients did not (HR 0.84; 95% CI 0.62–1.13; *p* = 0.26).

Our analysis has certain limitations. First, this was a retrospective, single-center analysis with multiple subgroups, our statistical results need to be interpreted in that light. Second, the GRS was originally investigated in ischemic patients with mild to moderate symptoms, which has differed from our cohort. Third, in our cohort, only CRT patients were presented, thus during the GRS calculation, the lowest value was 1 for all due to the wide QRS, which might influence our score analysis. Also, a selection bias can be presumed since only a limited percent of patients’ data could be analyzed. The analyzed patient groups differed significantly in mortality risk factors which might influence our results.

To conclude, in our retrospective single-center, large-scale, real-world clinical data, patients with non-ischemic HF who underwent CRT-D implantation did not acquire mortality benefit of having a defibrillator compared to CRT-P implantation. These results need further investigation; randomized trials are needed to assess and confirm our retrospective observations.

With the GRS physicians have at their hands an easily calculable risk stratification score in everyday clinical practice, made up of certainly assessed variables before device implantation. Selection of low and intermediate may help to achieve the most favorable outcome for non-ischemic HF patients. These patients may benefit the most from the addition of a defibrillator to CRT during long-term follow-up, whereas high-risk patients are unlikely to.

Our results have further clinical implications for non-ischemic patients, considering CRT-P implantation with optimal HFrEF medical treatment would be essential in those with very-high or low GRS. Besides calculating the GRS, the administration of drugs that showed clear evidence of risk reduction in mortality or SCD as sacubitril/valsartan or SGLT2 inhibitors may prolong or diminish the need for ICD implantation in CRT patients.

## Data availability statement

The original contributions presented in this study are included in the article/Supplementary material, further inquiries can be directed to the corresponding author.

## Ethics statement

The studies involving human participants were reviewed and approved by the Regional and Institutional Committee and Research; No. 161-0/2019. Written informed consent for participation was not required for this study in accordance with the national legislation and the institutional requirements.

## Author contributions

EM and WS participated in the conceptualization and designing of the study, in the data analyzation, and drafting of the manuscript. AB, LK, and BV engaged in data collection, participated in the interpretation of results, and thoroughly reviewed the manuscript. IO, RP, LM, EZ, and LG participated in the critical discussion of the data and reviewed the manuscript. AK made a major contribution to the conceptualization and execution of the study, supervised data collection, and critically reviewed the manuscript. BM supervised the study execution and thoroughly reviewed the manuscript. All authors read and approved the final version of the manuscript.
