# Supplementary material for: Predicting the survival benefit of cardiac resynchronization therapy with defibrillator function for non-ischemic heart failure—Role of the Goldenberg risk score
Source: Front Cardiovasc Med. 2023 Jan 10;9:1062094. doi: 10.3389/fcvm.2022.1062094 (PMC9871919; doi:10.3389/fcvm.2022.1062094)
Supplement: Supplementary file 1 [file Table_1.DOCX]

Supplementary materials:

Supplementary Table 1. Baseline clinical characteristics of the total patient cohort included in the study and the total non-ischemic HF patients underwent CRT implantation

| Characteristics | Total cohort  n=718 | Total non-ischemic HF patients  n=1290 | p-value |
| --- | --- | --- | --- |
| Age, years (median, 25^th^-75^th^ percentile) | 66 (59-73) | 66 (58-73) | 0.29 |
| Female sex, n (%) | 233 (32) | 408 (32) | 0.70 |
| Ejection fraction, % (median, 25^th^-75^th^ percentile) | 28 (23-32) | 28 (24-32) | 0.71 |
| QRS duration, ms (median, 25^th^-75^th^ percentile) | 160 (140-170) | 160 (140-176) | 0.14 |
| NYHA I, n (%) | 8 (1) | 4 (0.3) | **0.02** |
| NYHA II, n (%) | 299 (42) | 384 (30) | **‹0.001** |
| NYHA III, n (%) | 321 (44) | 446 (34) | **‹0.001** |
| NYHA IV, n (%) | 90 (12) | 139 (11) | 0.23 |
| Hypertonia, n (%) | 503 (70) | 854 (66) | 0.08 |
| Atrial fibrillation, n (%) | 286 (40) | 489 (38) | 0.39 |
| COPD, n (%) | 116 (16) | 188 (14) | 0.34 |
| Creatinine, mg/dl (median, 25^th^-75^th^ percentile) | 1.0 (0.9-1.1) | 1.0 (0.9-1.4) | 0.97 |
| BUN, mg/dl (median, 25^th^-75^th^ percentile) | 22.4 (17.4-30.5) | 22.1 (17.4-30.1) | 0.83 |
| ACE-I/ARB, n (%) | 631 (88) | 1056 (81) | **‹0.001** |
| Beta-blocker, n (%) | 618 (86) | 1011 (78) | **‹0.001** |
| MRA, n (%) | 470 (65) | 781 (60) | **0.03** |
| Loop diuretic, n (%) | 544 (76) | 895 (69) | **‹0.01** |
| Digoxin, n (%) | 148 (21) | 257 (20) | 0.71 |
| Amiodarone, n (%) | 197 (27) | 279 (22) | **‹0.01** |
| Mortality |  |  |  |
| Absolute rate, n (%) | 306 (48) | 629 (49) | 0.80 |

ACE-I (Angiotensin-converting enzyme inhibitor); ARB (angiotensin II receptor blocker); BUN (blood urea nitrogen); COPD (chronic obstructive pulmonary disease); CRT-D (cardiac resynchronization therapy- defibrillator); MRA (mineralocorticoid receptor antagonist); NYHA (New York Heart Association).

Supplementary table 2. Number of total patients based on their device type by the Goldenberg risk score.

| Goldenberg risk score | 1 | 2 | 3 | ›3 | VHR |
| --- | --- | --- | --- | --- | --- |
| All, n | 118 | 234 | 176 | 139 | 51 |
| CRT-P, n (%) | 47 (40) | 122 (52) | 93 (53) | 85 (61) | 34 (67) |
| CRT-D, n (%) | 71 (60) | 112 (48) | 83 (47) | 54 (39) | 17 (33) |

CRT-D (cardiac resynchronization therapy- defibrillator); CRT-P (cardiac resynchronization therapy-pacemaker); VHR (very-high risk)

Supplementary table 3. Absolute mortality rates based on the Goldenberg risk score.

| Goldenberg risk score | 1  (n= 118) | 2  (n= 234) | 3  (n=176) | ›3  (n= 139) | VHR  (n= 51) |
| --- | --- | --- | --- | --- | --- |
| CRT-P, n (%) | 15 (31.9) | 64 (52.4) | 52 (55.9) | 61 (71.7) | 27 (79.4) |
| CRT-D, n (%) | 12 (16.9) | 33 (29.5) | 28 (33.7) | 32 (59.3) | 13 (76.5) |
| Δ, (%) | -15 | -22.9 | -22.2 | -12.4 | 2.9 |
| p-value | 0.07 | ‹0.005 | 0.004 | 0.14 | 0.99 |

CRT-D (cardiac resynchronization therapy- defibrillator); CRT-P (cardiac resynchronization therapy-pacemaker); Δ (mortality difference); VHR (very-high risk)
